# Intensive 7-day internet-delivered cognitive behaviour therapy for social anxiety disorder: study protocol for a randomised controlled trial

**DOI:** 10.1186/s13063-025-08826-6

**Published:** 2025-04-18

**Authors:** Kayla R. Steele, Emily Upton, Monique Holden, Amy Regan, Matthew J. Coleshill, Sophie Li, Amy E. Joubert, Alison E. J. Mahoney, Michael Millard, Jill M. Newby

**Affiliations:** 1 School of Psychology, University of New South Wales, Sydney, Australia; 2 Black Dog Institute, University of New South Wales, Sydney, Australia; 3 UNSW Medicine & Health, University of New South Wales, Sydney, Australia; 4 The Clinical Research Unit for Anxiety and Depression, St Vincent’S Hospital, Sydney, Australia

**Keywords:** **S**ocial anxiety disorder, Anxiety disorders, Internet cognitive behavioural therapy, Internet- and mobile-based intervention, Intensive treatment, Cognitive behavioural therapy, Randomised controlled trial

## Abstract

**Background:**

Social anxiety disorder (SAD) is a serious mental health disorder that when left untreated can lead to significant social, occupational, educational, and functional impairment. Cognitive behaviour therapy (CBT) is the recommended first-line psychological treatment for SAD and has been shown to be efficacious in face-to-face and online formats. However, treatment is lengthy, and many people drop out prematurely. Pilot research suggests that brief intensive internet CBT (iCBT) for SAD is feasible and acceptable, but further evaluation using randomised controlled trials (RCT) is needed.

**Methods:**

A RCT to evaluate the acceptability, feasibility, and efficacy of an intensive 7-day iCBT program for adults diagnosed with SAD (with or without comorbid major depressive disorder) in comparison to a waitlist control (WLC) is currently in progress. Eligible participants will be randomised to an intervention or WLC group. Participants allocated to the intervention will complete 6 iCBT modules over 7 days, with CBT skills practice each day, and clinician guidance provided remotely by telephone and email. Participants allocated to the WLC will be given access to the program after a 6-week waiting period. All participants will complete assessments at baseline, 2 weeks and 6 weeks post-baseline. Outcome measures will assess for social anxiety (SIAS, SPS), depression (PHQ-9), personality (LPFS, PID-5-BF), and functioning (WSAS). Intention-to-treat linear mixed model analyses will be used to evaluate primary and secondary outcomes.

**Discussion:**

Previous findings from a pilot trial showed that 7-day iCBT is feasible and acceptable to clients with SAD. Based on these findings, we expect the treatment group will improve significantly on measures of symptoms of social anxiety, depression, and functional impairment compared to the WLC, and these improvements will be sustained at 1-month follow-up. If demonstrated to be effective in this RCT, intensive 7-day iCBT for SAD is a novel way to deliver CBT more quickly, with potential to reach more clients and reduce drop-out rates. It has great potential to provide rapid symptom improvement to individuals with SAD.

**Trial registration:**

This trial was prospectively registered with the Australian New Zealand Clinical Trials Registry on March 1, 2022 (ACTRN12622000361707).

**Supplementary Information:**

The online version contains supplementary material available at 10.1186/s13063-025-08826-6.

## Background

Social anxiety disorder (SAD) is a highly prevalent and often debilitating condition in which social interactions or being observed triggers intense fear or anxiety about being negatively evaluated or rejected by others [1]. SAD is estimated to impact approximately 14.4% of Australians between the ages of 18–85 over a 12-month period [2] and has been found to be more prevalent in women, particularly during adolescence [1]. Left untreated, SAD can lead to significant social, occupational, educational, and functional impairment for the individual [3], as well as social and economic impacts on wider society [4]. SAD has been found to be highly comorbid with other mental health conditions, particularly major depressive disorder (MDD), which SAD often precedes [5], and personality disorders, with co-occurrence of SAD and avoidant personality disorder estimated to range from 22 to 89% [6–8]. Notably, both comorbid depressive symptoms [9] and personality pathology [10] can exacerbate the severity of social anxiety symptoms and impact treatment response.

Clinical practice guidelines recommend cognitive behaviour therapy (CBT) as the preferred first-line treatment for SAD [11]. CBT has been shown to be efficacious when delivered in both face-to-face and online formats [11], with some studies finding that internet CBT (iCBT) is just as, if not marginally more, effective than face-to-face CBT, as well as more accessible [12, 13]. A systematic review [14] of over 20 randomised controlled trials (RCTs) found that iCBT (when delivered over 9 or 10 weeks) had a moderate positive average pooled between-group effect size (*g* = − 0.55) on patients’ social anxiety symptoms relative to control groups, increasing to a large pooled between-group effect size (*g* = − 0.79) when compared to a waitlist control group. Treating social anxiety symptoms using iCBT has also been found to have positive effects for treating comorbid MDD and depressive symptoms [15, 16].

Despite its efficacy, CBT for SAD is relatively lengthy, with a typical duration of 8–15 sessions over 10–16 weeks, and there are high drop-rates (an average of 18.3% for individuals with SAD; [17]). This means that many participants do not receive a full ‘dose’ of treatment and are at risk of poorer treatment outcomes [18, 19]. To address issues with treatment retention, CBT has been adapted to be successfully delivered in shorter or more intensive formats, in concentrated face-to-face sessions over 1–4 weeks, or in longer, more intensive sessions over 1–2 days. Promising effects for intensive CBT have been found for the treatment of perinatal anxiety [20], panic disorder [21, 22], and insomnia [23] in both face-to-face and iCBT modalities. Further meta-analyses have also found that intensive CBT and standard paced face-to-face CBT results in equivalent outcomes for anxiety and obsessive–compulsive symptoms, and may lead to greater improvement in depressive symptoms.

Some research studies have also found that intensive CBT can effectively treat SAD in a face-to-face, group-based format [24–28]. To the best of our knowledge, until our team conducted a pilot trial of intensive internet CBT, no studies had evaluated the efficacy of intensive CBT for SAD when delivered online and individually. Delivering intensive CBT treatment online individually may help overcome several barriers to accessing face-to-face treatment, including travel time, cost, appointment wait times, stigma, and clinician availability [29, 30], as well as the potential anxiety triggered by face-to-face contact with a clinician or a group-based setting [31]. The shorter lead time of an intensive treatment also means it has the potential to produce more rapid improvement in both symptoms [32] and functioning [33], which is often significantly impaired in people with SAD.

### 7-day iCBT for social anxiety disorder

We developed a novel intensive iCBT intervention for SAD, adapted from an existing evidence-based 6-module iCBT program for SAD. The program is typically delivered over 10–12 weeks [34–44] and available on www.thiswayup.org.au to individuals residing in Australia. The intensive delivery of the program was modelled on another pilot trial of an intensive exposure-based iCBT program for panic disorder and agoraphobia [45]. The 6 modules are delivered over a 1-week period (one module completed per day, with a rest day on day 6), and support is provided by a psychologist via telephone and email throughout the treatment week. Modules are approximately 20–30 min in length, and participants are encouraged to spend 3–4 h per day practising the assigned skills. For a description of the contents of the intervention see Table 1. The results of a small pilot trial conducted in 2020 with 16 participants with SAD [33] provided preliminary evidence of the intervention’s acceptability and feasibility, showing it significantly reduced participants’ social anxiety symptoms (Hedges’ *g*_*s*_ = 1.26–1.9), functional impairment (*g*_*s*_ = 0.88–0.98), and comorbid depressive symptoms (*g*_*s*_ = 0.88–0.98). The trial showed strong retention with 93.8% (15 out of 16) participants completing all modules; all participants rated the program as either very or mostly satisfactory.

**Table 1:** Intensive internet-based cognitive behaviour therapy for social anxiety disorder program overview

Module	Title	Description	Overview	Homework tasks	Follow-up
1	Social anxiety explained	Learn about how social anxiety affects you and how it can be treated	• Psychoeducation about social anxiety, CBT, and the ‘anxiety cycle’ • Setting goals for the program • Psychoeducation about the fight-or-flight response and arousal reduction strategies (e.g. controlled breathing, progressive muscle relaxation, physical exercise)	• Goal setting for the course • 30 min of exercise a day, 3 days per week • Practising controlled breathing 2–3 times per day • Completing guided progressive muscle relaxation	Day 1 telephone check-in call
2	Tackling avoidance	Understand how avoidance keeps anxiety going and how anxiety can be overcome using exposure therapy	• Psychoeducation about the role of avoidance and safety behaviours • Identifying one’s own avoidance and safety behaviours • Rationale for exposure therapy • Creating an exposure step ladder	• Identifying avoidance and safety behaviours • Creating an exposure step ladder • Confronting situation at the bottom of step ladder to begin with, then slowly moving up • Continuing with exercise, controlled breathing, and progressive muscle relaxation	
3	Trouble-shooting exposure	Review how exposure therapy is progressing and learn how to do behavioural experiments	• Reviewing and trouble-shooting exposure tasks • Using video feedback to test anxious predictions	• Identifying any pitfalls experienced with exposure • Selecting and implementing a solution for these pitfalls • Continuing to progress through exposure step ladder • Continuing with exercise, controlled breathing, and progressive muscle relaxation	Day 3 telephone check-in call
4	Unhelpful thinking styles	Learn to identify unhelpful thinking styles	• Introduction to the cognitive model and unhelpful thinking styles • Thought monitoring • Attention shifting	• Identifying relevant unhelpful thinking styles • Identifying times when worry is most prominent • Practising thought monitoring • Practising attention shifting • Continuing to progress through exposure step ladder • Continuing with exercise, controlled breathing, and progressive muscle relaxation	
5	Thought challenging and social skills	Challenge unhelpful thinking styles and learn about social skills	• Steps for thought challenging • Strategies for improving communication skills	• Practising thought challenging • Reading extra resources on conversation skills and assertiveness • Continuing to practise attention shifting • Continuing to progress through exposure step ladder • Continuing with exercise, controlled breathing, and progressive muscle relaxation	Day 5 telephone check-in call (optional)
6	Preventing relapse	Summarising the course and preventing relapses	• Psychoeducation about lapses and relapses • Relapse prevention planning • Identifying where to seek further support (if required)	• Continuing with program skills • Complete relapse prevention plan	

Despite these encouraging initial findings, further investigation is needed to evaluate the program’s efficacy. Because of the lack of control group in the pilot trial, it is unknown whether the improvements observed were attributable to the treatment or to another factor, such as the passage of time or repeated assessments. Further, the study relied on self-report measures of social anxiety. The lack of follow-up assessment also means it is unknown whether the gains observed were maintained over the longer term. A RCT with a larger sample, comparison to a control group, and inclusion of follow-up and clinician-rated assessment is now needed to evaluate the efficacy of the program.

A further important area of investigation is for whom intensive iCBT is best suited. While an intensive online format has potential for rapid symptom improvement, unwanted side effects (e.g. feeling overwhelmed from time pressure) have been reported by some participants in intensive iCBT (e.g. [20, 21, 33]), so better understanding of which patients may benefit most from this new treatment format is important. One study [46] of standard-paced iCBT for SAD for example found that older age and female gender predicted better treatment adherence and outcome, while another [47] found lower baseline severity predicted better post-treatment outcomes. A meta-analysis of 28 studies of standard-paced CBT for SAD however found no consistent evidence for strong pre-treatment predictors of response across the studies [48]. It is also unclear whether the efficacy of intensive iCBT is impacted by common comorbidities in SAD, such as depressive symptoms and personality pathology, which have been shown to predict poorer treatment response in standard-paced CBT [48, 49]. We therefore sought to investigate these questions further by examining predictors of treatment response and adherence to intensive iCBT for SAD, including demographic characteristics (age, gender) and comorbid psychopathology (baseline severity of depression and SAD symptoms, personality pathology).

### The current study

The proposed research will examine whether delivering iCBT in an intensive format over 1 week is effective in treating SAD symptoms relative to a waitlist control (WLC) group. A WLC condition was chosen as a comparator for several reasons. First, it provided a practical and feasible first step in evaluating the efficacy of this treatment, to determine whether a larger, more costly trial to compare it to an active treatment group or standard-paced CBT is warranted. WLC provided a clearer measure of treatment effects as it is more standardised than a usual care control group. Usual care control groups introduce more heterogeneity because participants receive varying levels and types of treatment. The WLC design also controlled for some factors such as the passage of time, the effects of repeated assessment, and regression to the mean which may have explained some improvements observed in the previous pilot trial. This study is the first RCT evaluating the efficacy of intensive, individual, online CBT for SAD. It is strengthened by the addition of follow-up assessment 1 month post-treatment and a clinician-rated diagnostic interview to assess the efficacy of the treatment beyond the self-report measures used in the pilot trial.

### Trial aims and hypotheses

This protocol has been reported according to SPIRIT guidelines for reporting of trial protocols [50, 51]. The primary aim of this study is to investigate the efficacy of the intensive iCBT for SAD program [44] in improving symptoms of social anxiety at post-treatment and 6-week follow-up, relative to a WLC group. The secondary objectives of the trial are to evaluate the efficacy of the program in reducing symptoms of depression and functional impairment/quality of life at post-treatment and follow-up. A third aim is to further explore the program’s acceptability and feasibility. We aimed to test whether the strong adherence and satisfaction observed in the pilot trial were replicated in a larger sample and outside of the COVID-19 pandemic context. A fourth aim is to explore predictors of treatment effectiveness, including participant age, gender, baseline symptom severity, and personality.

The primary hypothesis is that participants who receive the intensive iCBT for SAD intervention will show significantly reduced social anxiety symptom severity compared to the WLC group. We also hypothesise that intensive iCBT for SAD will be significantly more effective at improving participants’ comorbid depressive symptoms and increasing quality of life relative to the WLC group. The third hypothesis being tested is that intensive iCBT for SAD will be acceptable to participants according to high adherence (> 80%) and treatment satisfaction ratings. A further set of exploratory analyses will investigate whether the effectiveness of the intervention is predicted by participant age, baseline symptom severity, and personality functioning.

## Methods

### Trial design and setting

This superiority trial will utilise a two-arm, parallel group RCT design with an equal allocation ratio. Outcome measures will be assessed at baseline, post-intervention (primary endpoint, measured at 2 weeks post-baseline), and follow-up (secondary endpoint, measured at 6 weeks post-baseline). The primary time point of interest is post-treatment (2 weeks post-baseline). A 6-week follow-up was selected to ensure participants in the control condition were provided with the intervention after a reasonable duration and to reduce participant attrition.

This clinical trial is sponsored by the University of New South Wales (UNSW) and ethics approval has been granted by the UNSW Human Research Ethics Committee (HREC; HC#220,089). The trial has been registered with the Australian New Zealand Clinical Trials Registry on March 1, 2022 (ACTRN12622000361707).

### Eligibility criteria

Inclusion criteria includes adults aged 18 years or over, residing in Australia, fluency in written and spoken English, access to a computer and the internet, elevated scores on both the Social Interaction Anxiety Scale [> 34; SIAS, [52]] and the Social Phobia Scale [> 24; SPS, [52]], meeting DSM-5 diagnostic criteria for SAD according to a telephone diagnostic interview, willingness to provide basic demographic information and their general practitioner’s (GP) contact details, and on a stable dose of medications if applicable (no new medications or changes to existing medications in the 2 months prior to applying for the study).

Exclusion criteria are as follows: self-disclosed pre-existing diagnosis of schizophrenia, psychosis or bipolar disorder, daily or nearly daily suicidal ideation (score of ‘3’ on item 9 of the Patient Health Questionnaire-9 [PHQ-9; [53]]), or recent suicidality as determined by clinician interview (i.e. current suicidal intent, self-harm, or suicide attempts in the past 5 years), very severe depression (PHQ-9 scores of ‘24’ or above), currently receiving CBT, or commenced or changed mental health medication in the 2 months prior to application.

### Trial recruitment processes

**Fig. 1:**
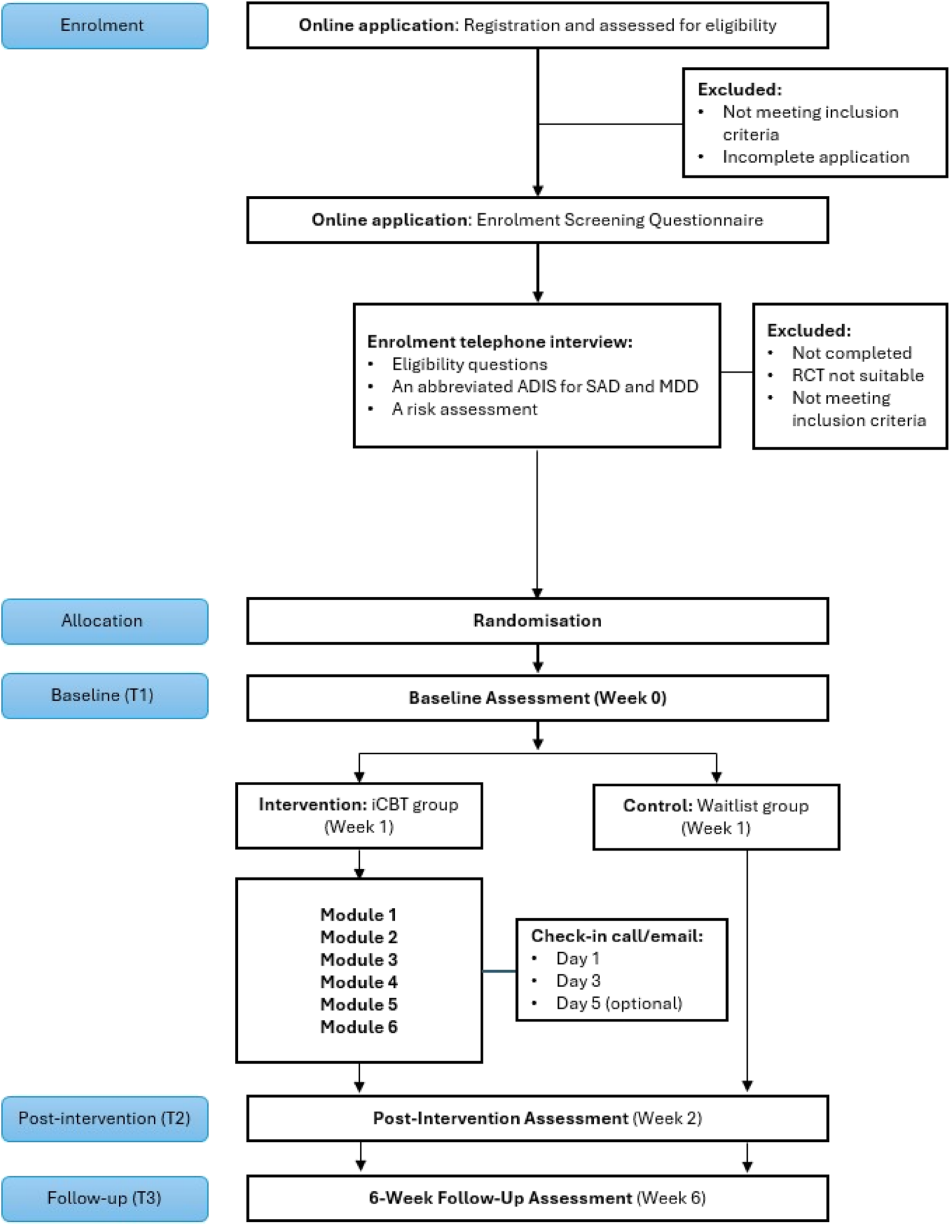
Consolidated Standards of Reporting Trials (CONSORT) participant flowchart illustrating enrolment and study procedure

The trial procedure and enrolment process is outlined in Fig. 1. This study is utilising an online recruitment strategy, with self-selecting participants recruited through paid study advertisements published on the Black Dog Institute website and social media channels (Facebook, Twitter, and Instagram), and through a research register of people with social anxiety who have consented to being contacted about research study participation. Applicants will be directed to the study website, where they can read information about the study, and click on a link to read the participant information statement and consent form (PISCF), administered by REDCap survey platform [54, 55]. To register, individuals provide electronic informed consent and are emailed a copy of the PISCF. Each time a participant logs in to the website to complete a lesson and/or questionnaire, this will be taken as a sign of continued consent to participate.

Applicants are next directed to complete an online screening and eligibility questionnaire, including demographic information, GP details, eligibility questions, and the SIAS, SPS, and PHQ-9. Ineligible applicants will be redirected on-screen and by email to further support services including digital mental health treatment options (e.g. the standard-paced version of the treatment through THIS WAY UP under supervision of their GP) and crisis support lines, and encouraged to speak with their GP about alterative support.

Eligible applicants will proceed to a phone call with one of the study clinicians (either a provisional psychologist, registered psychologist, or clinical psychologist) to complete additional eligibility questions, an abbreviated version of the Anxiety Disorders Interview Schedule for DSM-5 [ADIS; [56]] Social Anxiety Disorder and Major Depressive Disorder modules, and a comprehensive risk assessment.

Participants who meet inclusion criteria will be randomly allocated to one of two treatment groups (see randomisation and allocation procedures below) and verbally informed of their treatment group allocation and next steps. Participants’ general practitioners will be informed by letter of their participation and an outline of the intervention content.

Participants can self-withdraw their consent to take part in the study at any time by emailing the research team or submitting the UNSW standard withdrawal form, appended to the PISCF. Participants may also be withdrawn from the study if they experience a serious adverse event that is either attributable to the study intervention or procedure or that means they can no longer participate in the study.

### Trial monitoring and management

This study is coordinated and managed by the Black Dog Institute, a mental health research institute affiliated with UNSW, Sydney, based in Sydney, NSW, Australia. All clinician support and clinician-administered measures will be delivered via telephone and email by research clinicians based at the Black Dog Institute.^1^ All study procedures and the collection of participant data, including consent and outcome measures, will be administered via the REDCap survey platform [54, 55], which is hosted on secure servers commissioned by UNSW at GovDC Data Centres located in Sydney, Australia. Participant data is de-identified: the REDCap platform automatically generates a unique participant code which the research team will use to refer to participants to protect their confidentiality; and identifiable applicant and participant data will only be accessible to members of the research team. The data generated from REDCap is stored in a SQL Server 2016 database which is backed up daily. There will be no data monitoring committee or planned interim analyses.

For analyses, study assessment data will be exported from the REDCap platform via a Microsoft Excel file to SPSS version 26. These files will be password protected and stored on UNSW OneDrive. No identifiable information (e.g. name, date of birth) will be included in the main datafile used for analysis. This dataset will be stored using a password protected OneDrive file.

### Safety monitoring and management

This trial will follow a standardised risk management protocol based on previous clinical trials of digital mental health interventions run by the research team and use a Trial Management Group (TMG) to supervise the overall conduct and safety of the trial. This is in accordance with the National Health and Medical Research Council (NHMRC) guidelines on Data Safety Monitoring [57], which state that a TMG is an appropriate alternative to a Data Safety Monitoring Group for non-commercial trials. All adverse events and serious adverse events will be formally recorded and reported to the TMG, and in a report and to the UNSW HREC by the chief investigator. The TMG may recommend pausing or ending the trial due to participant safety concerns. UNSW can audit trial conduct at any time, and this process will be independent of the investigators.

To ensure the safety and wellbeing of participants, several methods are incorporated into the study design. First, all applicants will complete measures of suicidal ideation and depression symptom severity (i.e. PHQ-9). Second, eligible applicants will also complete a comprehensive phone-based risk assessment with a study clinician during the enrolment telephone interview. Any applicant who has recent history of self-harm, active suicidal intent, or recent suicide attempts will be ineligible for the study, and complete a phone-based safety plan, and provided referrals to alternative sources of crisis and ongoing support. Any applicant or participant who endorses suicidal ideation (without current intent) will complete a collaborative phone-based safety plan prior to their initial or ongoing participation; this safety plan will be emailed to them. If needed, further support services accessible after the study will also be discussed with participants. Participants will be emailed crisis support options in their orientation email, instructed to monitor for mood changes during trial participation, and contact the research team or their GP during business hours for non-emergencies, or call the state-wide crisis support teams or emergency numbers if in crisis.

Third, all participants will be actively monitored for mood deterioration, increase in suicide risk, or the experience of adverse side effects of the intervention in their questionnaire responses (PHQ-9, pre-module questions), during clinician check-in calls, and during the follow-up questionnaires and interview. Automated alert emails will be sent to the trial coordinator if participants score greater than ‘1’ on item 9 of the PHQ-9 at any time during the trial, indicating experience of suicidal thoughts several times over the past 2 weeks, and participants will be contacted by a study clinician the same or next business day to check in and complete a risk assessment and safety plan as needed. Participants will be provided the option to withdraw from the study if it is causing distress and referral options for a higher step of care will be discussed. Any presence of risk or changes in applicant or participant risk profile will be discussed amongst the TMG during weekly meetings and documented in the trial safety monitoring register.

### Intervention: 7-day iCBT for social anxiety disorder group

The intervention program follows the THIS WAY UP iCBT program for social anxiety (available on www.thiswayup.org.au), using the intensive 1-week format introduced in the pilot trial [33] and described further in Table 1. Participants allocated to the intervention group (iCBT for SAD group) will be able to choose their preferred start date for the intervention as early as the week after the phone interview. To streamline tracking of participants and shared clinical care between research clinicians during business hours, participants are scheduled to begin their treatment on a Monday and end the following Sunday. A preparation call following a standardised script will be scheduled for the week prior to a participant’s treatment week, reiterating treatment week instructions, discussing their treatment goals, scheduling check-in calls for days 1 and 3, and answering any questions prior to commencement.

On the first day of the treatment week, participants will receive an automated email from the REDCap platform notifying them that their first module is available, with a written record of instructions for the coming week. Participants will complete baseline questionnaires prior to the first module. Each day participants are instructed to spend 20–30 min reading module content, before downloading a brief summary and activity plan, and spending 3–4 h over the remaining day practising the assigned skills.

Following treatment completion, participants will receive an automated email via REDCap with a link to complete post-treatment (T2) and follow-up (T3) questionnaires. At 6 weeks (T3), participants will complete the telephone diagnostic interview (SAD and MDD modules).

### Control: waitlist control group

Participants allocated to the WLC group will be informed that they will be able to access the treatment after a waiting period of 6 weeks, during which they will complete three sets of online questionnaires. WLC participants will be sent automated emails via the REDCap platform with a link to complete questionnaires the day after their interview is completed (baseline, T1), 2 weeks later (T2), and 6 weeks post-baseline (T3). At T3, they will also be contacted to re-complete the telephone diagnostic interview, after which they will schedule the start date for their treatment week if they still choose to participate. WLC participants will follow the same procedure as the treatment group before (preparation call), and during their treatment week, receiving the treatment and clinician support over 1 week, and complete post-treatment questionnaires 1 week after completing their intervention. This post-treatment assessment will be used to assess WLC participants’ immediate treatment outcomes which will be included in the analysis of predictors of treatment outcome. The WLC will not be asked to complete any further follow-up assessments to minimise participant burden.

### Clinician support

Scheduled check-in calls will follow a standardised script. Questions are designed to prompt discussion with the participant around their understanding of the content and skills covered in the preceding modules, their goals, practice of the skills, and assistance troubleshooting any barriers or challenges to skills practice (e.g. tailoring an exposure hierarchy to their personal goals). Participants can request to complete their check-in calls via email if they are unable to speak over the phone (e.g. phone calls are too anxiety-provoking) or require less intensive support. If participants require further support over the following days, they can request a check-in call on day 5 of the program. Participants are informed that they may email the research team at any time during the treatment week with questions or if they require technical or clinical assistance (and will be responded to within business hours). All contact hours with participants will be recorded by clinicians in the REDCap platform.

### Randomisation and allocation procedures

At the completion of the telephone recruitment interview, participants will be randomised to either the iCBT for SAD group or WLC group using a 1:1 allocation ratio. Allocation numbers will be determined by a person who is completely independent of the research team, using the random.org random number generator and stored in opaque sealed envelopes, only opened by the study clinician once an offer of participation is made.

### Blinding

Treatment group allocation will be concealed from the research team and applicant until it is determined an applicant meets inclusion criteria and is invited to participate in the study. Treatment group allocation cannot be concealed from the participants or the researchers during group allocation, as participants will be informed by the researchers when they can commence their treatment. Primary outcome data will be based on self-report assessment to protect from experimenter bias. It is not feasible to conduct blinded diagnostic interviews at follow-up due to funding constraints.

### Trial outcomes and description of materials

After randomisation, all participants will complete a set of self-report questionnaires at baseline (T1), post-treatment (T2), and at 1-month follow-up (T3). Both groups will also be asked to complete a brief phone diagnostic interview [the ADIS MDD and SAD modules; [56]] at the 1-month follow-up (T3). Table 2 includes a list of assessment measures used and at which time point.

**Table 2:** Materials used at each study time point

Questionnaire	Recruitment (T0)	Baseline (T1)	Post-treatment (T2)	One-month follow-up (T3)
Demographics	X			
Eligibility questions	X			
ADIS-5 modules	X			X
SPS	X	X	X	X
SIAS	X	X	X	X
PHQ-9	X	X	X	X
WSAS	X	X	X	X
LPFS-BF 2.0		X		X
PID-5-BF		X		X
HSU	X			
CEQ		X		
TSQ^a^			X	
Strategy use			X	X

^a^TSQ is completed by the treatment group only

### Primary outcome measures

The co-primary outcome measures will be the Social Phobia Scale [SPS; [52]], a 20-item self-report measure of the respondent’s characteristic or typical fear of negative evaluation in social performance situations (e.g. public speaking, eating in front of others), and its companion measure, the Social Interaction Anxiety Scale [SIAS; [52]], a 20-item measure of anxiety related to social interactions (e.g. attending parties). These scales are frequently administered in tandem to comprehensively assess for both of these major components of social anxiety [58]. Both scales are widely used in clinical research, with studies demonstrating evidence of internal consistency (*α* = 0.89–0.94), test–retest reliability (*r* = 0.91–0.93) [52, 59], and construct validity, including concurrent validity, discriminant validity, and treatment sensitivity [52, 57–65].

### Secondary outcome measures

Patient Health Questionnaire-9 [PHQ-9; [53]]: The PHQ-9 is a 9-item, widely used, reliable and valid measure of depression symptom severity over the past 2 weeks [43, 66, 67]. Evidence of internal consistency (*α* = 0.86–0.92), test–retest reliability (*r* = 0.84), sensitivity and specificity (both 0.88), and construct, convergent, and discriminant validity, as well as treatment sensitivity have been reported [66–70].

Work and Social Adjustment Scale [WSAS; [71]]: The WSAS is a 5-item measure of quality of life and interference in the domains of work, home management, leisure activities, and close relationships. In clinical samples, evidence of internal consistency (*α* = 0.70–0.94), test–retest reliability (*r* = 0.73), convergent and discriminant validity, and treatment sensitivity has been provided [71–73].

Level of Personality Functioning Scale—Brief Form 2.0 [LPFS-BF 2.0; [74]]: The LPFS-BF 2.0 is a 12-item measure of level of personality functioning in the domains of self (identify and self-direction) and other (empathy and intimacy). It has demonstrated internal consistency (*α* = 0.82), discriminant validity, construct validity, and sensitivity to change over treatment, and has been validated in varying clinical, forensic, and community samples worldwide [74–78].

Personality Inventory for DSM-5—Brief Form [PID-5; [79]]: The PID-5-BF is a 25-item measure of the following personality trait domains: negative affect, detachment, antagonism, disinhibition, and psychoticism. For the each of the facets, internal consistency [*α* = 0.72–0.96 [79]], test–retest reliability (*r* = 0.57–0.87), construct validity, structural validity, and convergent validity have been demonstrated [80–84]. Both the LPFS-BF 2.0 and PID-5-BF were administered at baseline and 1-month follow-up only due to the impact of the relatively short time period on test–retest reliability and treatment sensitivity [67, 78], and to reduce participant burden.

Credibility/Expectancy Questionnaire [CEQ; [85]]: The first four items of the CEQ measures participants’ perception of the credibility and expected perceived benefit of a treatment before its commencement. It has been shown to possess internal consistency across several studies [*α* = 0.85–0.90; [85–87]], as well as temporal stability, and predictive validity for treatment outcome [85].

Bespoke questions about health service use (HSU): Including participants’ current psychotropic medication use, previous experience of treatment for anxiety or depression, and level of experience with CBT.

### Additional outcome measures

Participants in the treatment group will be administered additional questionnaires to assess the acceptability, treatment satisfaction, treatment engagement, feasibility, and side effects of the intervention.

Module feedback: Prior to starting each lesson, participants will be asked to provide brief feedback about the previous module with the question ‘Do you have any feedback (positive or negative) about the previous lesson that you would like to share with us? Please be honest as it helps us to improve the program’.

Strategy use: Participants will be asked about the frequency with which they have used each strategy (e.g. ‘using video feedback’ or ‘thought challenging’; from 0 = never to 5 = very often), and an open-ended question asking which strategy they feel was most helpful in reducing their fear of social situations.

Treatment Satisfaction Questionnaire [TSQ; [88]]: the TSQ is a 5-item measure of treatment satisfaction used frequently in RCT studies evaluating CBT treatments [e.g. [89–91]]. It prompts participants to rate on a series of Likert scales their overall level of satisfaction with the treatment, the pace of the treatment, how logical it seemed, how confident they were that the program was successful in teaching them skills to manage their anxiety, and how confident they would be in recommending the treatment to a friend with similar symptoms.

Side effects: At post-treatment, participants will be asked to describe any positive effects or events and any unwanted side effects or negative events they feel occurred because of the program. Prior to starting each module, participants will also be asked if they experienced any difficult or adverse events since the last lesson.

Further open-ended questions will be included in post-treatment questionnaires regarding participants’ treatment experiences, including which aspects of the treatment they found most helpful and unhelpful and that they liked and disliked the most; how the program could be improved; how relatable they found the characters; how the characters could be improved to be more relatable to them; and any further comments.

Further qualitative questions regarding participants’ experience of the program will be asked in the 1-month follow-up interview, including their overall experience of using the program; their opinion of the strategies suggested; whether there were any other topics they would have liked covered in the program; any suggested changes to the program; what they liked most and least about the program; and any further feedback. These questions are included to ensure participants have an opportunity provide their feedback and provide suggestions on how to improve the program. This information will be used to inform refinements and improvements to the program and treatment model before any further studies are conducted.

We will measure adherence using rates of program completion (i.e. the total number of modules completed out of 6) and engagement by asking participants to self-report approximately how many minutes they spent reading the previous module materials and how many minutes they spent practising the skills in the previous day. These questions are also asked at the conclusion of module 6. Further qualitative questions around engagement in the 1-month follow-up interview will be asked, including whether participants continue to use any strategies from the program (and which ones); any changes in behaviours that have occurred since using the program; what contributed to their continued engagement; and any barriers to engagement that they experienced and suggestions for reducing these barriers.

### Statistical analysis

All analyses will be undertaken using intention-to-treat approach. Linear mixed models, with a random intercept for subject will be used to analyse primary and secondary outcomes, and covariances matrixes used to assess relationships between variables. This approach will be used to manage missing data and account for correlation between repeated measures. Before conducting the analyses, patterns of missing data will be assessed through data visualisation, and Little’s Missing Completely at Random (MCAR) test to determine whether the data are missing at random. If there are significant group differences (as determined by independent samples *t*-tests) in the amount of time elapsed between the intake interview and completion of baseline measures, this variable will be included as a covariate in the linear mixed models. Planned contrasts will be used to compare pre- and post-treatment, and pre-treatment and 1-month follow-up, and planned pairwise comparisons will be used to compare between-groups at post-treatment and follow-up. To control for the risk of type 1 error with a co-primary outcome, the alpha level for significance will be adjusted using a Bonferroni correction (alpha set at 0.025 to determine statistical significance for the SIAS and SPS co-primary outcome measures). Linear mixed models will be utilised to test the predictors of treatment outcome. Within- and between-group effect sizes will be calculated using Hedges’ *g* (with 95% confidence intervals) to estimate the within-group changes from baseline to post-treatment, and baseline to follow-up, and between-group differences on primary and secondary outcome measures at post-treatment and follow-up.

### Sample size calculations

The planned sample size is 50 participants, with participants randomly allocated to either a treatment group (*n* = 25) or a waitlist control group (*n* = 25). Power calculations are based on an estimated between-group effect size of 0.92 based on a meta-analysis of iCBT compared to controls [92] with alpha set at 0.05 and power at 0.8. A minimum sample of 20 participants per group is required to detect this effect size. An additional 5 participants per group will be recruited to account for expected attrition based on our pilot trial [33].

### Dissemination

When data analysis is finalised, a summary of research findings will be emailed to all participants and made available on the Black Dog Institute website. The research study will also be prepared for publication in relevant peer-reviewed scientific journals and presented at academic conferences. In all papers, numerical data will be aggregated, and no individual participants will be identifiable. All qualitative data will also be non-identifiable.

### Lived experience input

During the planning of this study, the investigator team consulted with a small group (*n* = 5) of members of the Black Dog Institute Lived Experience advisory network. Lived experience advisors provided recommendations regarding the preferred name of the program, the timing and format of the clinician support, the preparation process, and the advertising material, as well as the choice, type (e.g. qualitative, and quantitative), and focus of the assessment measures.

## Discussion

The aim of our trial is to investigate the efficacy, acceptability, and feasibility of an intensive 1-week iCBT program for SAD compared to a waitlist control. To the best of our knowledge, this is the first RCT evaluating the efficacy of an intensive individually delivered 7-day iCBT treatment for any disorder including SAD. While our pilot trial [33] demonstrated preliminary evidence for the acceptability, feasibility, and positive outcomes of the intervention, including large reductions in social anxiety symptoms and functional impairment, the current study aims to extend these results using a more rigorous two-arm parallel-group RCT with follow-up.

The inclusion of a WLC group in this trial will allow for stronger conclusions to be made about treatment effects beyond external factors such as the passage of time, treatment expectancy, or clinician contact during the recruitment phase. The addition of clinician-rated measures and a diagnostic interview will enable us to assess the effects of the program on diagnoses of SAD and depression further to the self-report measures used in the pilot trial. Using validated clinical structured diagnostic interviews to assess SAD at baseline and follow-up will allow for stronger conclusions around intervention outcomes as they are less influenced by social desirability biases than self-report measures [93].

The study is strengthened by the inclusion of various outcome variables that explore the impact of the intervention beyond social anxiety symptoms, including on depression, quality of life, functional impairment, and personality traits and functioning. The inclusion of 1-month follow-up measures will also allow us to ascertain if treatment outcomes are sustained after the intensive 1-week treatment period.

The current study further extends the pilot trial as it is being completed outside of COVID-19 pandemic conditions. Significant increases in the use of online interventions for anxiety and depression were seen during the pandemic [94]. This study will allow us to ascertain whether the strong results and high levels of engagement found in the pilot trial are replicated in the current, post-pandemic period.

The inclusion of extensive qualitative open-ended questioning around participants’ experience of the treatment, with a direct focus on feedback around treatment satisfaction, side effects, treatment preferences, and suggestions for improvement, is a further strength of this study. The feasibility and acceptability of intensive iCBT is not well understood, and consequently, this qualitative data will help us to make evidence-based, patient-centred improvements to the treatment as needed before wider dissemination outside of research settings.

### Limitations of the study and future research

The results of this study should be interpreted with consideration of some limitations. Firstly, the use of a passive (waitlist) versus active control group means that while it is discouraged for participants to commence other therapies or medication during the waiting period, we are unable to control for other care they receive during this time. Future studies would benefit from comparing the treatment to an active control condition such as a digital placebo program or a psychoeducation control group. Future research should also compare this intervention to standard-paced iCBT or intensive face-to-face CBT. While our predictor analyses will provide some initial understanding as to whom this intensive approach is most suited, a RCT comparing the intensive and standard-paced formats (and to traditional face-to-face formats) will provide further clarification as to who benefits most from these diverse treatment approaches. A further limitation relates to the relatively short follow-up period; future studies would be strengthened by a longer-term follow-up period (e.g. 3 months or longer) to measure the longer-term impact of the intervention. In addition, our power calculations have been informed by meta-analyses of standard paced iCBT [92] and may have overestimated the expected between-group effect size. Future studies should consider using minimal important clinical differences to inform sample size calculations, to ensure similar studies are powered to detect changes that are not only statistically significant, but clinically meaningful.

Second, the risk of bias cannot be completely eliminated in the collection of some study data. It is not feasible to blind researchers to participant group allocation follow-up interviews. We will also rely on participant self-report to measure some engagement variables (length of time spent reading module material or practising skills) so this may be inaccurate. Relatedly, as the treatment is completed by participants online rather than in vivo with a clinician, it is not possible for clinicians to model the therapy skills (e.g. exposure). As such, it will be unclear if participants are implementing these skills with complete fidelity to the program instructions.

A further limitation relates to the generalisability of the results given that the sample of included participants will be self-referred and likely motivated to complete the trial. The results we obtain in this trial may not generalise to all individuals with SAD but rather to a subset who are interested, engaged, and motivated to try an intensive treatment approach. Future studies should test the generalisability and effectiveness of the program outcomes in a community sample, in routine-care settings, which tend to draw a more diverse and representative sample of participants including those with more severe and complex presentations [e.g. [95, 96]].

Finally, for reasons of feasibility, clinical support will be shared amongst the clinical team over a participants’ treatment week. It will be unclear whether this has an impact on participants during their treatment. If feasible, consistency in the clinician providing clinical support to each participant may be a consideration for future research trials.

## Conclusion

Intensive face-to-face CBT and iCBT have consistently demonstrated efficacy for improving symptoms of SAD. However, to the best of our knowledge, no studies have evaluated the efficacy of intensive individual iCBT for SAD delivered online. Intensive online treatments have the potential to help overcome barriers to accessing traditional face-to-face treatments by reducing costs, having immediate availability, and not relying on the regular and ongoing availability of trained clinicians, which is particularly valuable in rural and remote areas. If demonstrated to be effective, intensive iCBT for SAD may be a novel way to deliver treatment faster, with potential to reach more clients, and reduce drop-out rates. We believe it has great potential to provide rapid symptom improvement for individuals with SAD. Considering these potential benefits, investigating the efficacy of intensive iCBT for the treatment of SAD is an important area of future research and studies that adopt rigorous methodologies (including RCT with follow-up) are much needed.

## Trial status

This trial was registered with the Australian New Zealand Clinical Trials Registry on March 1, 2022 (ACTRN12622000361707). Trial recruitment began on March 18, 2024, and the first trial participant was enrolled on March 26, 2024. The trial protocol was submitted for publication in May 2024. Recruitment was completed in June 2024 and all data collection finalised by the end of July 2024, with analysis of results currently underway.

## Supplementary Information

Additional file 1: SPIRIT checklist.

## Data Availability

After publication of the study results, de-identified data may be made available to other researchers upon reasonable request.

## Abbreviations

ADIS-5: Anxiety Disorders Interview Schedule for DSM-5
CBT: Cognitive behavioural therapy
CEQ: Credibility/Expectancy Questionnaire
iCBT: Online cognitive behavioural therapy
LPFS-BF 2.0: Level of Personality Functioning Scale—Brief Form 2.0
PHQ-9: Patient Health Questionnaire-9
PID-5-BF: Personality Inventory for DSM-5 Brief Form
SAD: Social anxiety disorder
SIAS: Social Interaction Anxiety Scale
SPS: Social Phobia Scale
TSQ: Treatment Satisfaction Questionnaire
WLC: Wait list control
WSAS: Work and Social Adjustment Scale

## Footnotes

1 As Australian states share time zones within a few hours of one another, telephone support from clinicians in NSW is accessible nationwide.
